# Predictors of early versus late mortality in pelvic trauma patients

**DOI:** 10.1186/s13049-016-0220-9

**Published:** 2016-03-10

**Authors:** Hao Wang, Richard D. Robinson, Billy Moore, Alexander J. Kirk, Jessica Laureano Phillips, Johnbosco Umejiego, Joseph Chukwuma, Tyler Miller, Donna Hassani, Nestor R. Zenarosa

**Affiliations:** Emergency Department, JPS Health Network, 1575 S. Main St, Fort Worth, TX 76104 USA; Research Institute, JPS Health Network, 1500 S. Main St, Fort Worth, TX 76104 USA; University of South Florida, Morsani College of Medicine, 12901 Bruce B. Downs Blvd., MDC061, Tampa, FL 33612-4799 USA

**Keywords:** Pelvic trauma, Mortality, Time-related, Predictors

## Abstract

**Background:**

Risks of predicting time-related in-hospital mortality varies in pelvic trauma patients. We aim to identify potential independent risks predictive of time-related (early versus late) mortality among pelvic trauma patients.

**Methods:**

Local trauma registry data from 2004 through 2013 were reviewed. Mortality causes and timing of death were investigated. Multivariate logistic regression identified independent risks predictive of early versus late mortality in pelvic trauma patients while adjusting for patient demographics (age, sex, race), clinical variables (initial vital signs, mental status, injury severity, associated injuries, comorbidities), and hospital outcomes (surgical interventions, crystalloid resuscitations, blood transfusions).

**Results:**

We retrospectively collected data on 1566 pelvic trauma patients with a mortality rate of 9.96 % (156/1566). Approximately 74 % of patients died from massive hemorrhage within the first 24 h of hospitalization (early mortality). Revised trauma score (RTS), injury severity score (ISS), initial hemoglobin, direct transfer to operating room, and blood transfusion administration in the Emergency Department were considered independent risk factors predictive of early mortality. Age, ISS, and Glasgow Coma Scale (GCS) were deemed risk factors predictive of death after 24 h (late mortality).

**Discussion:**

Given the fact of a substantial number of patients died within the first 24 h of hospital arrival, it is reasonable to consider the first 24 h of hospitalization as the appropriate window within which early mortality may be expected to occur in pelvic trauma patients. The risk factors associated with massive hemorrhage were strong predictors of early mortality, whereas late mortality predictors were more closely linked with comorbidities or in-hospital complications.

**Conclusions:**

While risk factors predictive of early versus late mortality vary, ISS seems to predict both early and late mortality accurately in pelvic trauma patients.

## Background

Pelvic trauma is one of the most severe and complicated types of injury and is associated with relatively high mortality and morbidity [1–3]. Different independent risk factors predictive of in-hospital mortality in pelvic trauma have been reported. Results from a United Kingdom pelvic trauma registry study showed that patient age, initial blood pressure, mental status, injury severity score, and other associated injuries were independent risk factors for mortality [4]. Other international studies have not only confirmed these findings, but have also affirmed other risk factors predictive of in-hospital mortality in pelvic trauma such as whether patients received packed red blood cell transfusions within the first 12 h (h) of hospitalization and whether the patient was injured intentionally [5, 6]. Several North American studies have asserted other in-hospital mortality predictors among pelvic trauma patients, such as low initial hemoglobin and direct transfer to the operating room (OR) [7–12]. Regardless of different risks predicting in-hospital mortality in pelvic trauma patients, the most common independent risk factor in the literature is severity of injuries as determined via revised trauma score (RTS) and injury severity score (ISS) systems [13, 14].

In addition, literature also reported predictors vary with in-hospital mortality at different time points. Chong et al. [10] reported the majority of pelvic trauma patients whose deaths occurred within the first 72 h were due to pelvic hemorrhage, non-pelvic injury, or brain trauma, while those deaths occurring later (>72 h) were attributed to multisystem organ failure or acute respiratory distress syndrome (ARDS). Gunst et al. [15] reported different risks occurred in early (≤4 h) versus late (>4 h) mortality in pelvic trauma patients noting higher risk of late mortality in this group. Other evidence has also shown low hemoglobin and blood product administration are common early mortality predictors, whereas age is a common and consistent predictor of late mortality [12, 16–18]. Severity of injuries scores including RTS and ISS, although valuable in predicting severity, do not effectively predict *time-related* mortality among pelvic trauma patients. Previous studies have shown that both RTS and ISS accurately identify severity of traumatic injuries and predict mortality in general [19–21]. Other tools have determined different values of predicting time-related mortality in pelvic trauma with either RTS or ISS [12, 22, 23]. Furthermore, though these studies reported the value of incorporating RTS and ISS in the prediction of early versus late hospital mortality their definitions of early mortality were different from other studies [19, 24]. Since in-hospital mortality predictors among pelvic trauma patients varies especially in the prediction of time-related mortality, it is important to differentiate early versus late in-hospital mortality, delineate its etiologies, and identify its appropriate predictors associated with time.

Generally, ‘early’ in-hospital mortality refers to patient death occurring within the first 24 h of hospital arrival while any death occurring beyond this point is considered ‘late’. This is partly due to the diverse causes of death in pelvic trauma [7, 18]. Early in-hospital death among pelvic trauma patients may directly or indirectly occur because of massive hemorrhage, whereas surgical complications, infections, or multisystem organ failure may occur after 24 h and relates to ‘late’ in-hospital death [7, 18, 25–27]. Though different ‘early’ in-hospital mortalities were reported, it appears that no such study focused on the validation of defining ‘early’ versus ‘late’ hospital mortality. Given the fact that in-hospital complications may occur within the first 48 to 72 h of hospitalization [11, 28] and pelvic trauma patients hospitalized longer than 24 h may still have potential for significant active bleeding [11, 28, 29, 30], it is worthwhile to raise the question as to whether it is suitable to consider 24 h as a reliable discrimination point for early death in pelvic trauma patients. In addition, further identification and validation of factors capable of accurately predicting early versus late mortality in patients suffering pelvic trauma is necessary to increase physician awareness of patients’ conditions and guide appropriate management.

Therefore, in order to better understand the causes of death and potential risks predictive of time-related mortality in pelvic trauma patients, we aimed to: 1) study what is a suitable window for early in-hospital mortality; 2) determine the common causes of early mortality; and 3) validate the different independent risk factors to predict early versus late mortality.

## Methods

### Selection of participants

Retrospective review of all patients entered into the local trauma registry during the period January 1, 2004 through December 31, 2013 was performed. The study included all adult (18 and over) patients with traumatic pelvic injuries that presented to the ED with signs of life (i.e., those not pronounced dead prior to or immediately on arrival to ED). Since this study focused on identifying independent risk factors associated with early versus late in-hospital mortality, patients missing final disposition data or for whom we were unable to identify survival status were excluded.

### Study design and protocol

For the purpose of this study, mortality refers to all cause in-hospital mortality. We did not set out to identify the direct causes of death due to pelvic injuries; instead we focused on common causes of in-hospital mortality among pelvic trauma patients reported in the literature, including massive hemorrhage, other associated injuries, and severe in-hospital or trauma related complications. Because death due to massive hemorrhage rarely occurred beyond the first 72 h of hospital admission, for this study, we defined mortality due to massive hemorrhage as such if any one of the following criteria were met: 1) patient required more than an initial 10 units blood transfusion (either packed red blood cell or whole blood) and subsequently required additional blood transfusion until death or the 72 h end-point was reached; 2) persistent hypotension despite continuous blood transfusions with continued down-trending hemoglobin; or 3) immediate transfer to operating room (OR) upon arrival to the hospital to facilitate exploratory laparotomy and hemorrhage control to include packing procedure (intra-operative damage-control approach).

Mortality was determined to be due to other associated injuries if hemorrhage, which was associated with injuries apart from pelvic trauma, was controlled (e.g. intra-abdominal organ injury and significant intra-abdominal bleeding was controlled) or the causes of massive hemorrhage were ruled out. In addition, patients meeting the following criteria were included in this group: 1) associated injuries confirmed by adjunct exams after hospital admission; 2) initial clinical presentation inconsistent with pelvic injuries; or 3) condition neither changed nor improved (e.g., mortality due to associated head injury was considered if patient did not meet hemorrhage criteria, initial presentation was altered mental status with confirmed intracranial pathology, and clinical condition worsened after hospital admission). Mortality classification could simultaneously encompass more than one associated injury if certain criteria were met. If a patient died before the completion of adjunct exams and treating physicians were unable to identify the associated injuries, an extensive chart review was conducted. If any of the following evidence (such as physician progress notes, consultant notes, imaging testing and lab results) were recorded in the patient’s medical record indicating, but not limited to: infection, acute respiratory distress syndromes (ARDS), pulmonary embolism, single or multisystem organ failure, the cause of death was categorized as secondary to in-hospital or trauma related complication(s). Again, two or more causes of death could occur in one patient simultaneously.

Mortality was divided into four categories according to time, within the first 24, 48, 72, or beyond 72 h of hospitalization. Causes of death were reviewed and determined independently by two physicians, each with ample trauma experience who are actively engaged in the management of trauma patients. If a discrepancy occurred, the study’s Principle Investigator acted as the third reviewer rendering a final decision. Inter-observer agreement was measured.

Based on the results of mortality analysis, a discrimination point for early mortality was identified. Patients were then divided into two groups (early mortality [E group] and late mortality [L group]). Basic patient demographics (age, sex, race, mode of arrival) and ED clinical variables (initial vital signs, RTS, GCS, associated injuries, amount of crystalloid received, blood transfusion administration) were analyzed and compared. In order to determine the potential risk factors predictive of early versus late in-hospital mortality, stepwise multivariate logistic regression analysis was performed. To determine whether early mortality in pelvic trauma patients could extend to 72 h post admission, dependent outcome variables (24 h, 48 h, and 72 h mortalities) were again used in multivariate logistic regression analysis. Their accuracy and discrimination levels were compared. The local institutional review board approved this study.

### Data analysis

Student’s *t* Test was used to compare continuous variables between two groups, while Pearson Chi-square (*χ*2) analysis was used to compare categorical variables. Inter-reader agreement testing was performed to determine the level of consistency among reviewers by using the kappa statistic (κ > 0.4, moderate agreement and κ > 0.6, strong agreement). In order to identify independent risk factors predictive of early versus late mortality and to avoid potential confounders, clinical variables were entered into a stepwise multivariate logistic regression model. After preliminary data analysis, it was found that some variables had missing values for 0.4 to 6.2 % of cases (Appendix). These missing data demonstrated an arbitrary pattern. Therefore, an iterative Markov Chain Monte Carlo (MCMC) method was used to impute the missing data in a multivariate normal model. Hosmer-Lemeshow test was performed to determine the goodness of fit for logistic regression model. C-statistic was used to assess the discriminatory power of the model. Models consisted of strong predictors if the C-statistic value was greater than 0.8. Receiver operating characteristic (ROC) curves were drawn and the area under the ROC (AUC) measurements were derived to determine the accuracy of models in predicting early versus late mortality. All descriptive and statistical analyses were performed using Stata 12.0 (College Station, TX). A *p* value less than 0.05 was considered statistically significant.

## Results

**Fig. 1:**
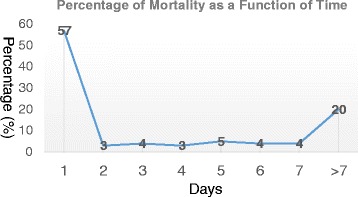
Percentage of Mortality as a Function of Time among All Deceased Pelvic Trauma Patients

A total of 1566 pelvic trauma patients met inclusion criteria. General patient information and clinical variables are reported in Table 1. Of those included, 156 died after admission, resulting in a total mortality rate of 9.96 %. Among all non-survivors, 57 % died within the first 24 h of admission and 20 % died after 7 days of hospitalization (Fig. 1). A total of 101 patients died within the first 72 h and 89 within the first 24 h of hospital arrival. Of those who died within the first 24 h, 74 % (66/89) were due to massive hemorrhage (Table 2). A significantly low percentage of patient deaths were due to in-hospital or trauma related complications (Table 2). Investigator reviewed deaths due to hemorrhage and associated injuries resulted in strong inter-reader agreement scores (κ = 0.70, *p* < 0.01 and κ = 0.74, *p* < 0.01). The kappa coefficient of agreement on patient death due to in-hospital or trauma related complication was 0.59 indicating a reasonable level of agreement (*p* < 0.01).

**Table 1:** General Pelvic Trauma Patient Information

Patient Demographics	
Age (Mean Years, SD)	42 (18)
Sex (Male, *n*, %)	1015 (65)
Race	
White (*n*, %)	1008 (64)
African American (*n*, %)	174 (11)
Hispanic (*n*, %)	293 (19)
Others^a^ (*n*, %)	91 (6)
ED Clinical Variables	
Systolic BP (Mean mmHg, SD)	122 (31)
Heart Rate (Mean, SD)	94 (24)
Respiratory Rate (Mean, SD)	18 (5)
GCS (Mean, SD)	12.9 (4.0)
RTS	
(Mean, SD)	7.15 (1.54)
(Median, IQR)	7.84 (0)
ISS	
(Mean, SD)	20 (13)
(Median, IQR)	17 (18)
Crystalloid Received at ED (Mean ml, SD)	1836 (1463)
Type of Injury	
Blunt (*n*, %)	1520 (97)
Penetrating (*n*, %)	46 (3)
Type of pelvic injury	
Ilium (*n*, %)	234 (15)
Ischium (*n*, %)	61 (4)
Pubis (*n*, %)	702 (45)
Sacrum/coccyx (*n*, %)	429 (27)
Acetabulum (*n*, %)	715 (46)
Unclear (*n*, %)	109 (7)
Associated Injuries (*n*, %)^b^	734 (47)
Head (*n*, %)	339 (22)
Chest (*n*, %)	215 (14)
Abdomen (*n*, %)	281 (18)
Extremity (*n*, %)	313 (20)
Blood Transfusion at ED (yes, %)	166 (11)
Hgb (Mean g, SD)	12.8 (2.4)
Transfer Directly to OR (yes, %)	275 (18)

*Abbreviations*: *SD* standard deviation, *BP* blood pressure, *GCS* Glasgow Coma Scale, *RTS* revised trauma score, *IQR* interquartile range, *ISS* Injury Severity Score, *ED* Emergency Department, *Hgb* hemoglobin, *OR* operating room

^a^: others include Asian, Pacific Islander, and Native American. ^b^: Associated injuries: injuries other than these four regions are not reported in this study

**Table 2:** Causes of Mortality at Specific Time Intervals in Pelvic Trauma Patients

Hospital Arrival	Hemorrhage	Associated Injuries	Complications^a^
0–24 h	66 (74 %)	43 (48 %)	3 (3 %)
24–48 h	3 (60 %)	4 (80 %)	0 (0)
48–72 h	6 (86 %)	3 (43 %)	1 (14 %)
>72 h	1 (2 %)	35 (64 %)	48 (87 %)

0–24 h: including 24 h; 24–48 h: including 48 h, not including 24 h; 48–72 h: including 72 h, not including 48 h; ^a^complications refer to in-hospital or trauma related complications as addressed in detail in Methods section

**Fig. 2:**
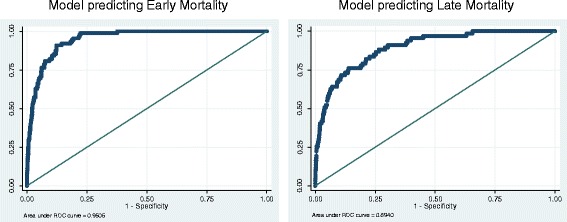
ROC Curves of Models Predicting Early and Late Mortality in Pelvic Trauma Patients

In order to determine independent risk factors predictive of in-hospital mortality, patients were divided into two groups. Patients who died within the first 24 h of arrival were placed in the ‘early group’ or E group, whereas those who died beyond 24 h were placed in the ‘late group’ or L group. Missing data were then imputed. The results of the Hosmer-Lemeshow goodness of fit test for logistic regression showed that the model fit the data well and the risk prediction was well calibrated (*p* = 0.34). Basic demographics and clinical information are listed in Table 3. L group tended to be older, have higher systolic blood pressure, heart rate, and hemoglobin, and received lower volume crystalloid and blood products resuscitation in the ED. Severity of injury, sex, race, mental status, associated injuries, and whether emergent surgical intervention was required, showed no statistically significant difference between groups (Table 3). Early versus late mortality predictive risk factors are reported along with their C-statistics (Table 4). ROC curves of models predicting early and late mortality are shown in Fig. 2. We found no statistically significant difference in independent risk factors among patients who died within 24, 48, or 72 h of hospital arrival. The C-statistics in three models predicting early mortality within 24, 48, and 72 h of hospitalization were 0.9505, 0.9501, and 0.9493 respectively (*p* > 0.05).

**Table 3:** Comparisons of General Information in Pelvic Trauma Patients: Early versus Late Mortality

	E group (*n* = 89)	L group (*n* = 67)	*P* value
Patient Demographics			
Age --- year, mean (SD)	44 (18)	51 (21)	<0.05
Gender ---Male, yes (%)	78	70	0.30
Race			
White (%)	68	75	<0.05
African-American (%)	17	6	
Hispanic (%)	16	12	
Others^a^ (%)	0	7	
Clinical Variables upon Patient Arrival at ED			
Systolic BP ---mmHg, mean (SD)	74 (50)	107 (37)	<0.01
Heart Rate ---bpm, mean (SD)	87 (52)	102 (34)	0.05
Respiratory Rate --- rpm, mean (SD)	13 (10)	15 (6)	0.12
GCS --- number, mean (SD)	6.67 (4.96)	7.95 (5.27)	0.13
RTS			
(Mean, SD)	3.93 (2.84)	5.41 (2.09)	<0.01
(Median, IQR)	4.09 (5.22)	5.97 (3.75)	0.12
ISS			
(Mean, SD)	37 (14)	36 (14)	0.63
(Median, IQR)	34 (18)	34 (14)	1.00
Crystalloid Received at ED ---ml, mean (SD)	2678 (1790)	2502 (2114)	0.61
Type of injury			
Blunt (%)	94	97	0.43
Penetrating (%)	6	2	
Location of pelvic injury			
Ilium ---*n*, (%)	12 (18)	9 (15)	0.60
Ischium---*n*, (%)	2 (3)	3 (5)	0.59
Pubis ---*n*, (%)	40 (59)	36 (58)	0.93
Sacrum/coccyx ---*n*, (%)	19 (29)	13 (21)	0.31
Acetabulum ---*n*, (%)	28 (41)	26 (42)	0.93
Unclear --- *n*, (%)	22 (25)	5 (7)	<0.01
Associated Injuries ---*n*, (%)	88 (99)	65 (97)	0.40
Head ---*n*, (%)	43 (48)	40 (60)	0.16
Chest ---*n*, (%)	55 (62)	40 (60)	0.79
Abdomen ---*n*, (%)	51 (57)	37 (55)	0.80
Extremities ---*n*, (%)	57 (65)	39 (59)	0.47
Blood transfusion at ED (yes, %)	52	34	<0.05
Hgb ---g, mean (SD)	9.7 (2.7)	11.9 (4.4)	<0.01
History of Cardiovascular Diseases (yes, %)	11	13	0.67
Transfer Directly to OR (yes, %)	49	39	0.19

*Abbreviations*: *E group* refers to pelvic trauma patients experiencing early death, *L group* refers to pelvic trauma patients experiencing late death, *SD* standard deviation, *BP* blood pressure, *bpm* beats per minute, *rpm* respirations per minute, *GCS* Glasgow Coma Scale, *RTS* revised trauma score, *IQR* interquartile range, *ISS* Injury Severity Score, *ED* Emergency Department, *Hgb* hemoglobin, *OR* operating room

^a^percentages may not add up to 100 % due to rounding up. Others includes Asian, Pacific Islander, and Native American

**Table 4:** Independent Risk Factors Predictive of Early versus Late Mortality in Pelvic Trauma Patients using Multivariate Logistic Regression Analysis

	Adjusted Odds Ratios (95 % CI)	C-statistics
Early Mortality		
Blood Transfusion at ED	2.14 (1.19–3.86)	0.72
Transfer to OR from ED	2.15 (1.21–3.81)	0.67
ISS	1.04 (1.02–1.06)	0.85
RTS	0.60 (0.53–0.69)	0.87
Hgb	0.73 (0.66–0.82)	0.82
Final Model		0.95
Late Mortality		
Age	1.05 (1.03–1.06)	0.63
ISS	1.08 (1.05–1.11)	0.85
GCS	0.84 (0.78–0.89)	0.81
Final Model		0.89

*Abbreviations*: *CI* confidence interval, *OR* operating room, *ISS* injury severity score, *RTS* revised trauma score, *Hgb* hemoglobin, *GCS* Glasgow Coma Scale

## Discussion

High mortality rates occur among patients with severe trauma, especially among those with pelvic injuries [1]. Previous studies have reported increased early mortality rates in pelvic trauma patients primarily due to massive hemorrhage and severity of traumatic injuries. Other studies have reported that late mortality among patients with pelvic injuries may be due to surgical complication, infection, and/or multisystem organ failure [4, 10, 31]. Given the fact that, in this study, a substantial number of patients died within the first 24 h of hospital arrival and the majority of them were due to hemorrhage, it might be appropriate to consider the first 24 h as a suitable discrimination point for early mortality. Risk factors predictive of early mortality occurred in pelvic trauma patients with less accuracy beyond the first 24 h [19;23]. Independent risk factors varied in the prediction of early versus late mortalities. The risk factors associated with massive hemorrhage were strong predictors of early mortality, whereas other factors were more closely linked with comorbidities or in-hospital complications. While severity of injury can predict in-hospital mortality, it appears in this study only ISS accurately predicts both early and late mortality in pelvic trauma patients. Our findings are consistent with the majority of previous studies focusing on pelvic trauma but with the added emphasis on identifying time-related mortality and its predictive risk factors [4, 7, 12]. Identifying potential time-related mortality risk factors will alert physicians to take appropriate early actions thus improving outcomes in pelvic trauma patients.

Previous studies identified early death with similar causes extending up to 72 h post admission [5, 10]. Very few studies, however, reported the percentage of early deaths separately at 24 h increments along with causes of death [11]. Those who did report time-related mortality showed the majority of early deaths occurred in the first 24 h of hospital admission and their causes of death were mostly associated with massive hemorrhage [7, 16]. Our study demonstrates similar findings while also comparing patients who died within 24, 48, and 72 h of hospital admission. Though a small percentage of patients died beyond 24 h from massive bleeding, it did not affect the accuracy of early mortality prediction in our study.

Mortality prediction without consideration of the timeline in which it occurred may not provide accurate results among pelvic trauma patients. When prediction is narrowed to either early or late mortality, less variety occurs. Our findings are consistent with the literature suggesting that risks associated with bleeding, such as initial hemoglobin, initial blood product administration, and severity of injury, are reliable independent risk factors predictive of early mortality, while age and associated injuries are reliable predictors of late mortality [4, 12, 28]. Some studies found that severe head injuries are predictors of early death while other studies suggest that patients with severe head injuries tended to die after 24 h of hospitalization [5, 10, 23, 32]. Our findings showed no significant difference in terms of mortality associated with these injuries. RTS was shown to predict early mortality whereas GCS predicted late mortality alone. This indicates that GCS should heavily weigh in on late mortality prediction in pelvic trauma patients. We are not certain, however, whether low GCS is directly linked to severity of head injuries in this study as this was not specifically investigated.

Both RTS and ISS are predictive of early mortality, though only ISS is predictive of late mortality. RTS is calculated based on patient GCS, blood pressure, and respiratory rate, which are closely related to hemodynamic stability. We therefore conclude this to be an accurate predictor of early mortality [14, 33]. ISS is determined by associated injuries and is calculated utilizing an abbreviated injury scale in each of the 6 body regions which summarizes the patient’s general condition in addition to the severity of the injuries within each region [34]. ISS not only identifies current injury severity but also estimates severity of associated injuries in pelvic trauma patients. As there is often a high rate of associated complex injuries in pelvic trauma, ISS becomes a reasonable variable to predict mortality [23, 35]. Our study validated the incorporation of ISS in predicting both early and late mortality. With the popular use of electronic medical documentation systems, it is now practical to calculate ISS by computer thereby providing real-time guidance to emergency physicians and trauma surgeons with respect to patient dispositions.

### Limitations

Retrospective study designs cannot demonstrate causality due to limited information accuracy, missing data and potential selection bias. The direct cause of death cannot be determined precisely without performing an autopsy in each patient and physician judgement, while determining cause of death, might lead to subjective bias. It is, however, unrealistic to autopsy every study patient to more accurately determine the cause of death. Therefore, all-cause in-hospital mortality was the outcome measurement in this study. Furthermore, two physicians conducted intensive chart reviews independently. This approach reduced the risk of bias. We were unable to include all clinical variables for mortality analysis such as in-hospital medical or surgical complications, patient baseline comorbidities, or prehospital transportation times, which could render predictions less accurate. Future prospective studies focusing on the validation of these risk factors as predictive of early and late mortality in pelvic trauma patients are warranted.

## Conclusions

It is reasonable to consider the first 24 h of hospitalization as the appropriate window within which early mortality may be expected to occur in pelvic trauma patients. The risks predictive of early versus late mortality are different. Patient initial hemoglobin level, patient RTS and ISS, emergent blood transfusion, and emergent surgical intervention are independent risk factors for early mortality. Patient age, ISS, and mental status on arrival are independent risk factors for late mortality. Overall, ISS seems to predict both early and late mortality well in pelvic trauma patients.

## Appendix

**Table 5:** Missing Data Rate of Variables used in this Study

Variables	Missing Data (*N*)	Missing Rate (%)
Age	0	0
Gender	0	0
Race	0	0
Mechanisms of Injury (Blunt/Penetrating)	0	0
Receiving Blood Transfusion at ED	0	0
Transfer to Operating Room	0	0
Injury Severity Score	6	0.4
ED Pulse	21	1.3
ED Systolic Blood Pressure	24	1.5
ED Glasgow Coma Scale	60	3.8
ED Respiratory Rate	65	4.2
Hemoglobin	97	6.2
Revised Trauma Score	97	6.2

*Abbreviation*: *N* number, *ED* Emergency Department
